# Feed nutritional composition affects the intestinal microbiota and digestive enzyme activity of black soldier fly larvae

**DOI:** 10.3389/fmicb.2023.1184139

**Published:** 2023-05-24

**Authors:** Guozhong Chen, Kai Zhang, Wenli Tang, Youzhi Li, Junyi Pang, Xin Yuan, Xiangbin Song, Linlin Jiang, Xin Yu, Hongwei Zhu, Jiao Wang, Jianlong Zhang, Xingxiao Zhang

**Affiliations:** ^1^School of Life Sciences, Ludong University, Yantai, China; ^2^Shandong Provincial Key Laboratory of Quality Safety Monitoring and Risk Assessment for Animal Products, Ji'nan, China; ^3^Yantai Key Laboratory of Animal Pathogenetic Microbiology and Immunology, Yantai, China; ^4^Shandong Breeding Environmental Control Engineering Laboratory, Yantai, Shandong, China

**Keywords:** black soldier fly larvae, food waste, nutritional composition, intestinal microbiota, microbial diversity, digestive enzymes

## Abstract

**Introduction:**

Using black soldier fly larvae (BSFLs) to treat food waste is one of the most promising environmental protection technologies.

**Methods:**

We used high-throughput sequencing to study the effects of different nutritional compositions on the intestinal microbiota and digestive enzymes of BSF.

**Results:**

Compared with standard feed (CK), high-protein feed (CAS), high-fat feed (OIL) and high-starch feed (STA) had different effects on the BSF intestinal microbiota. CAS significantly reduced the bacterial and fungal diversity in the BSF intestinal tract. At the genus level, CAS, OIL and STA decreased the *Enterococcus* abundance compared with CK, CAS increased the *Lysinibacillus* abundance, and OIL increased the *Klebsiella*, *Acinetobacter* and *Bacillus* abundances. *Diutina*, *Issatchenkia* and *Candida* were the dominant fungal genera in the BSFL gut. The relative abundance of *Diutina* in the CAS group was the highest, and that of *Issatchenkia* and *Candida* in the OIL group increased, while STA decreased the abundance of *Diutina* and increased that of *Issatchenkia*. The digestive enzyme activities differed among the four groups. The α-amylase, pepsin and lipase activities in the CK group were the highest, and those in the CAS group were the lowest or the second lowest. Correlation analysis of environmental factors showed a significant correlation between the intestinal microbiota composition and digestive enzyme activity, especially α-amylase activity, which was highly correlated with bacteria and fungi with high relative abundances. Moreover, the mortality rate of the CAS group was the highest, and that of the OIL group was the lowest.

**Discussion:**

In summary, different nutritional compositions significantly affected the community structure of bacteria and fungi in the BSFL intestinal tract, affected digestive enzyme activity, and ultimately affected larval mortality. The high oil diet gave the best results in terms of growth, survival and intestinal microbiota diversity, although the digestive enzymes activities were not the highest.

## Introduction

A large amount of food waste has caused serious environmental pollution (Sun et al., 2018), but it is difficult to handle (Abdel-Shafy and Mansour, 2018). Waste treatment technologies mainly include anaerobic digestion, heat-moisture reaction, composting, incineration and landfill disposal, which often lead to secondary pollution (Gao et al., 2017). In recent years, insect-based methods for the treatment of organic solid waste have received extensive attention, especially those based on black soldier fly (BSF, *Hermetia illucens*) (Sheppard et al., 1994). Compared with fly maggots and earthworms, black soldier fly larvae (BSFLs) have the advantages of diverse feeding habits, large food intake, high stress resistance and lack of disease transmission (Sheppard, 1983; Bradley and Sheppard, 1984; Sheppard et al., 1994; Erickson et al., 2004; Liu et al., 2008). BSFLs can feed on poultry manure and kitchen waste (Salomone et al., 2017; Rehman et al., 2023) and produce high-value animal protein feed (Bondari and Sheppard, 1987). Insect body fat can be used to produce biodiesel (Li et al., 2011), and insect manure can replace commercial fertilizer (Choi et al., 2009). Therefore, this method has become popular worldwide.

The developmental stages of BSF include the egg, larva, pupa and adult stages. Diet affects the hatchability of eggs, the size and mortality of larvae, the duration of the larval and pupal stages and the sex ratio and determines the physiological and morphological development of adults (Gobbi et al., 2013; Harnden and Tomberlin, 2016). Interestingly, diet affects not only the growth and development of BSF but also the gut microbiota (Jeon et al., 2011). The diet is considered a major driver for changes in gut bacterial diversity that may affect its functional relationships with the host (Ley et al., 2008). Different gut microbiota has different gene contents to adapt to different dietary nutrient acquisition strategies (Kurokawa et al., 2007). Tanga et al. (2021) found that different feed ingredients affect the weight gain of BSFLs and, importantly, lead to the transfer of intestinal microorganisms of BSFLs and changes in the bacterial community, while the fungal community is highly dependent on substrate. The composition of the intestinal microbiota of BSF under different diets deserves attention because some bacteria are beneficial to BSF (Bruno et al., 2019) but harmful to animals and humans (Khamis et al., 2020). For example, *Providencia* species in BSF are vertically transmitted bacteria that enhances oviposition (Smet et al., 2018), while in humans, they cause gastroenteritis, urinary tract infections, and other nosocomial infections in immunocompromised patients (Galac and Lazzaro, 2011). In addition, the intestinal microbiota of BSF plays an important role in substrate degradation and insect development (Jiang et al., 2019; Kooienga et al., 2020).

BSFLs can digest a variety of organic materials more efficiently than any other known fly species, which is directly due to the abundant digestive enzymes in its gut, including amylase, protease and lipase (Kim et al., 2011). Previous work has shown that changes in diet will cause modifications of the digestive enzymatic machinery of BSFLs, but it is not certain that rich nutrition will improve the digestive enzyme activity. It was found that low protein diet actually leaded to an increase in proteolytic activity, which may be due to the motion compensatory mechanisms initiated by BSFLs to make the best use of this rearing substrate (Bonelli et al., 2020). In addition to diet, the intestinal environment and intestinal compartment are also related to digestive enzyme activity (Espinoza-Fuentes and Terra, 1987), which may be attributed to the role of gut microbiota. In this paper, 16S/its amplicon sequencing technology was used to study the effects of high-fat, high-protein and high-starch diets on the intestinal flora of BSFLs, analyze the correlation between the intestinal microbiota and digestive enzyme activity, and compare the growth and development of larvae under feeding with different diets. The purpose of this study was to provide a theoretical basis for optimizing the nutritional ratio of BSF diet, and to further promote the engineering application of BSF in the treatment of food waste.

## Materials and methods

### Sample collection and preparation

Four diets, namely, the high-protein (CAS), high-oil (OIL), high-starch (STA) and control (CK) diets, were prepared as described in Table 1. As a basic dietary material, wheat bran contains 4.8% fat, 14.9% protein, 28.5% carbohydrates, and 33.9% dietary fiber, which can provide the nutrients needed for the growth and development of BSFLs. One thousand 3rd instar BSFLs were raised in a 2 L plastic box that was sterilized with 75% alcohol and covered with sterile gauze. Five replicates were set for each group. Feed supplementation was performed after disinfection according to the consumption behavior of the larvae. When approximately half of the larvae in a group had prepupated, feeding was stopped. BSFLs from the various substrates were collected, and the survival rate was calculated. For 10 randomly selected larvae, the body weight was recorded on a precision balance, the body length was measured with a Vernier caliper, and the average values were calculated. Then, the larvae were surface sterilized with 70% ethanol for 1 min and washed with sterile saline for 2 min. The entire gut of each larva was dissected aseptically using forceps and placed in a 2 ml microcentrifuge tube. One part of the sample was removed to measure the enzyme activity, and the other part was stored at −80°C until DNA extraction.

**Table 1:** Composition of the experimental diets.

Ingredient %	CK	CAS	OIL	STA
Wheat bran	63	63	63	63
Sawdust	37	23	17	14
Casein	0	14	0	0
Starch	0	0	0	23
Soybean oil	0	0	20	0

### Tissue preparation and enzyme assays

Intestine samples were homogenized in cold sodium phosphate buffer (0.1 M, pH 7.0, 4°C) at a ratio of 1:9 (m/v) in an icebox. Each sample was centrifuged at 4°C and 3,000 × g for 10 min, and the supernatant was collected and analyzed for digestive enzyme activity. The total protein content was determined using bovine serum albumin as the standard according to the methods of Bradford (1976). The *α*-amylase, *β*-amylase, pepsin, trypsin, chymotrypsin and lipase activities were evaluated using corresponding assay kits (Nanjing Jiancheng, Bioengineering Institute, China) (see Supplementary material for specific methods).

### DNA extraction and PCR amplification

Total DNA was extracted from the samples using the E.Z.N.A.^®^ Soil Kit (Omega Bio-tek, Norcross, GA, United States) according to the manufacturer’s instructions. The DNA concentrations and purities were measured using a NanoDrop 2000 device. The integrity of the extracted DNA was checked by 1% agarose gel electrophoresis. The V3-V4 variable regions of the 16S rRNA gene of the bacteria were amplified by polymerase chain reaction (PCR) using the primers 338F (5¢-ACTCCTACGGGAGGCAGCAG-3¢) and 806R (5¢-GGACTACHVGGGTWTCTAAT-3¢). The ITS1-ITS2 spacers of the fungal ribosomal genes were amplified by PCR using the primers ITS1F (5¢-CTTGGTCATTTAGAGGAAGTAA-3¢) and ITS2R (5¢-GCTGCGTTCTTCATCGATGC-3¢). TransStart FastPfu DNA Polymerase (catalog number AP221-02, TransGen Biotech) was used to perform PCR with the 338F and 806R primers. The reaction mixtures included 4 μL of 5× FastPfu buffer, 2 μL of 2.5 mM dNTPs, 0.8 μl of each primer (5 μM), 0.4 μL of FastPfu polymerase, 0.2 μL of bovine serum albumin (BSA), and 10 ng of DNA template. These ingredients were mixed together with double-distilled water to obtain a total reaction volume of 20 μL. TaKaRa rTaq DNA Polymerase was used to perform PCR with the ITS1F and ITS2R primers. The reaction mixtures included 2 μL of 10× buffer, 2 μL of 2.5 mM dNTPs, 0.8 μL of each primer (5 μM), 0.2 μL of rTaq polymerase, 0.2 μL of BSA, and 10 ng of DNA template. These reaction components were also mixed together with double-distilled water to obtain a total reaction volume of 20 μL. The PCR experiments were performed using the ABI GeneAmp^®^ 9,700 PCR system.

PCR products were recovered from a 2% agarose gel, purified using the AxyPrep DNA Gel Extraction Kit (Axygen Biosciences, Union City, CA, United States), eluted in Tris–HCl, and retested by 2% agarose gel electrophoresis. The results were quantified using a QuantiFluor^™^-ST fluorometer (Promega, United States). The purified fragments were used to construct a PE 2*300 library according to the standard operating protocol of the Illumina MiSeq platform (Illumina, San Diego, United States). Finally, the MiSeq PE300 platform (Illumina, Inc.) was used to perform sequencing. The raw data were uploaded to the NCBI database (SRA accession: PRJNA925287).

### Data analysis

The data were analyzed using a free online platform, namely, the MajorBio i-Sanger cloud platform.^1^ The original sequence data were subjected to quality-control processing using Trimmomatic software and then spliced using FLASH software as follows: (1) first, a 50-base pair (bp) window was established. If the average quality value in the window was lower than 20, then bases at the front end of the window were removed, resulting in a sequence length of 50 bp after quality control, (2) Overlapping sequences were spliced when the maximum mismatch rate was 0.2 and the overlap length was greater than 10 bp, and (3) The sequences were split into separate samples based on the barcode and primer sequences at the beginning and end of each read, respectively. We required an exact match with the barcodes, whereas a mismatch of 2 bases was allowed for the primers. In addition, sequences with fuzzy bases were removed.

All sequences were clustered into operational taxonomic units (OTUs) based on 97% similarity using UPARSE software (version 7.1 http://drive5.com/uparse/). Single sequences and chimeras were removed during the clustering process. Each sequence was classified by species using the RDP classifier^2^ and compared with the Silva database (SSU123), with an alignment threshold of 70%. SPSS version 16.0 (SPSS, Chicago, Illinois, United States) was used for statistical analysis of the soil microbial community diversity and relative richness. Specifically, all calculations were performed on replicate values, and analysis of variance was also performed. The average of 3 replicates was used for paired t test analysis. A *p* value of <0.05 was considered to reflect a statistically significant difference.

## Results

### Larval performance

**Figure 1:**
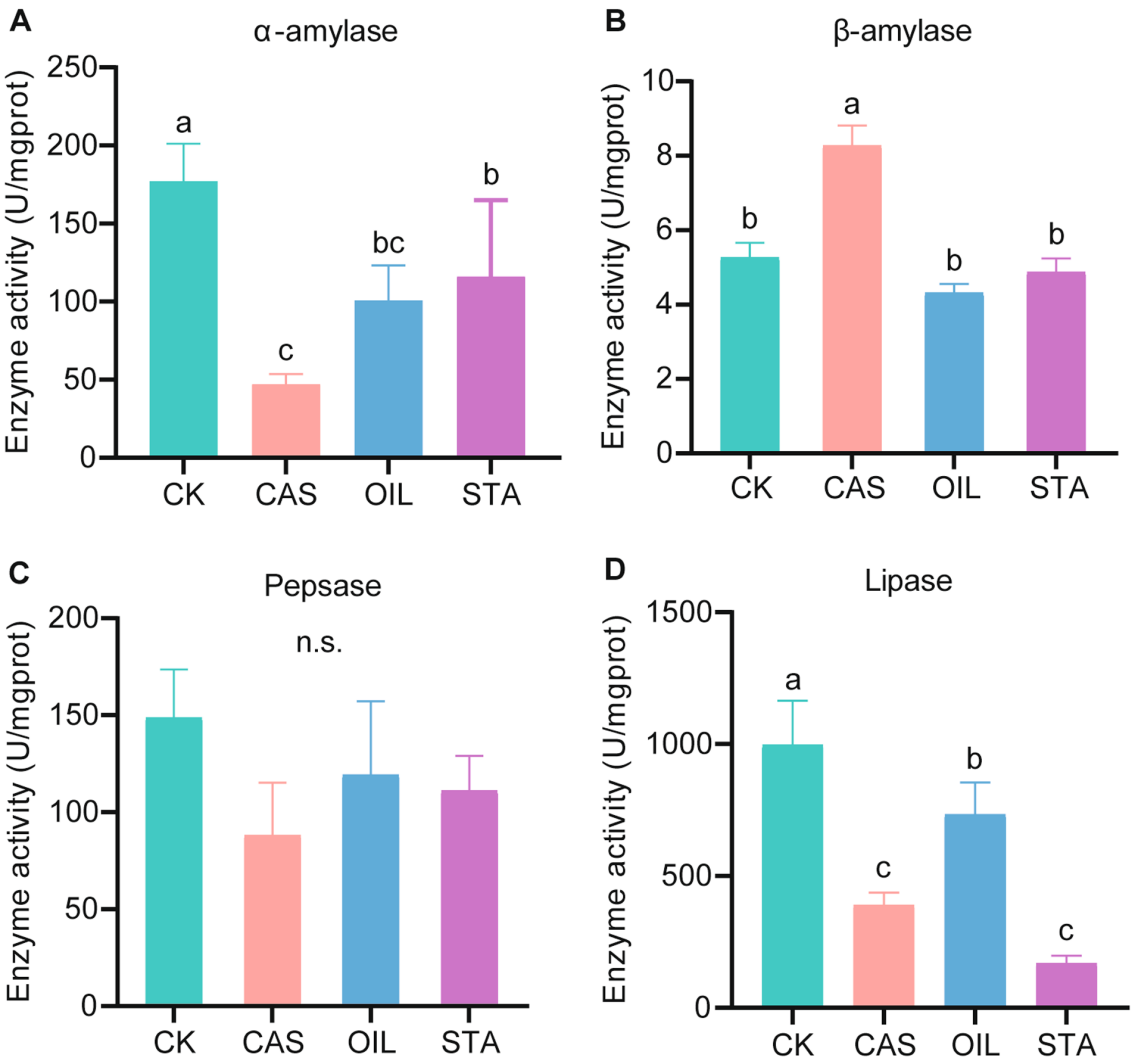
Digestive enzyme activity assay. α-Amylase-specific activity **(A)**, *β*-amylase-specific activity **(B)**, pepsase-specific activity **(C)** and lipase-specific activity **(D)** from the gut of BSFLs fed different diets. Error bars indicate standard deviation. Different letters on the columns indicate significant differences between groups at the *p* < 0.05 level. n.s. = not significant.

The digestive enzyme activities of the intestinal tract of BSF fed with different nutrient compositions were significantly different (Figure 1). The CK group had the highest α-amylase activity (177.13 U/mgprot), followed by the STA, OIL and CAS groups. The activity of β-amylase in the CAS group was the highest (8.28 U/mgprot), and there was no significant difference among the other three groups. The pepsase activity of the CK group (148.93 U/mgprot) was the highest and that of the CAS group (88.21 U/mgprot) was the lowest, but there was no significant difference among the four groups. The CK group had the highest lipase activity (998.73 U/mgprot), followed by the OIL, CAS and STA groups. Interestingly, except for *β*-amylase, the activities of the other three enzymes were the highest in the CK group and the lowest or nearly the lowest in the CAS group. In addition, the activities of trypsin and chymotrypsin were not detected.

**Figure 2:**
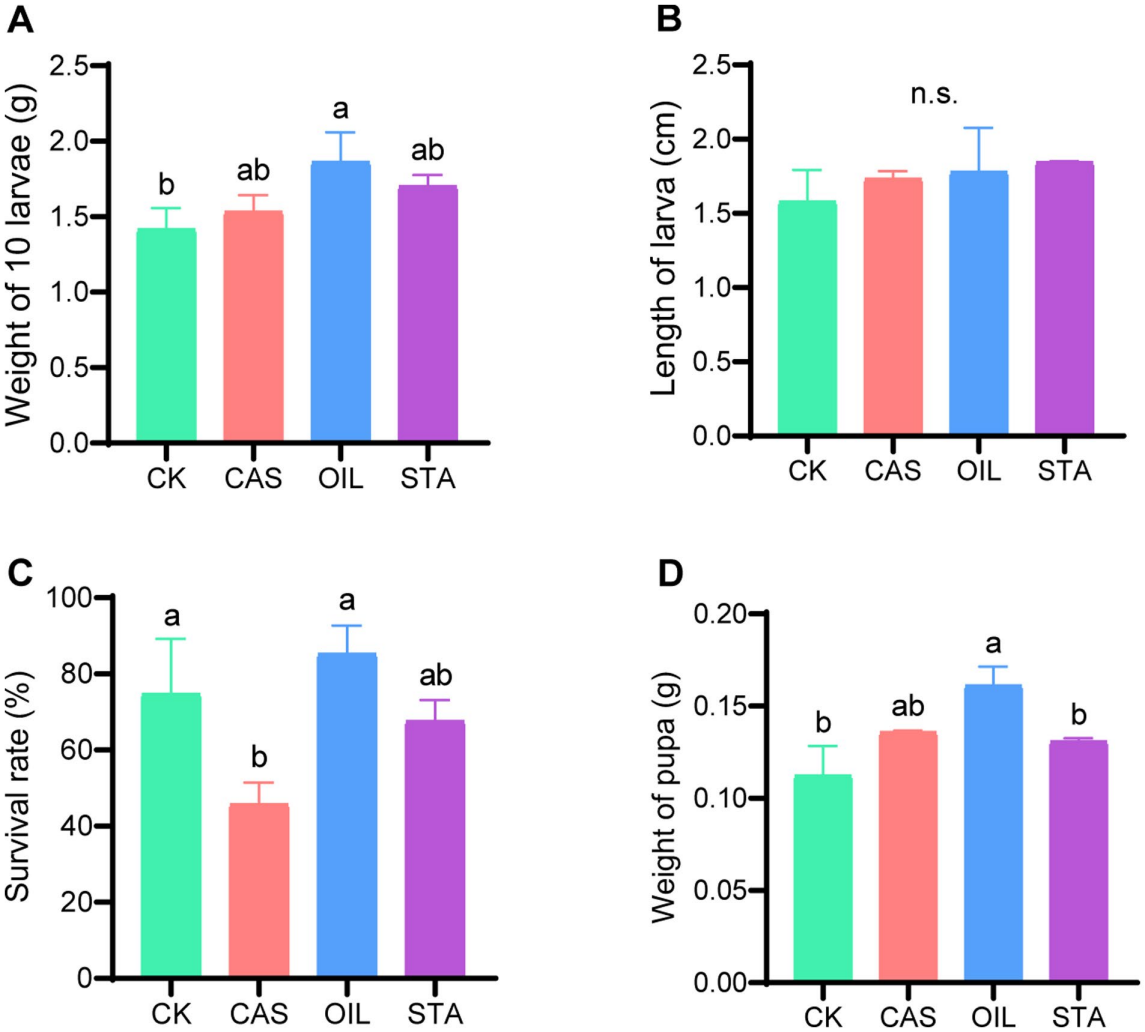
Larval performance. **(A)** Weight of 10 larvae; **(B)** length of larvae; **(C)** survival rate; **(D)** weight of pupae. Error bars indicate standard deviation. Different letters on the columns indicate significant differences between groups at the *p* < 0.05 level. n.s. = not significant.

The larvae in the OIL group were the heaviest (1.86 g/10 larvae) and were significantly heavier than those in the CK group (Figure 2A). The larvae in the CAS and STA groups were heavier than those in the CK group, but the difference was not significant. The larvae in the treated groups were longer than those in the CK group, although the difference was not significant (Figure 2B). The survival rate of larvae in the CAS group (45.83%) was significantly lower than that in the CK and OIL groups, and the latter had the highest survival rate (85.25%) (Figure 2C). The pupae in the OIL group were also the heaviest (0.16 g) and were significantly heavier than those in CK groups, while those in the CAS and STA groups were slightly heavier than those in the CK group, but there was no significant difference (Figure 2D). In summary, the high oil diet improved the performance of larvae.

### Richness and diversity of the microbial community

To explore the diversity of the intestinal microbial community of BSFLs fed different diets, 16S and ITS sequencing was performed using Illumina high-throughput sequencing technology. After filtering out low-quality reads and trimming adapters and barcodes, 1,093,106 effective bacterial sequences and 1,990,073 effective fungal sequences were generated from the microbial populations of four sample types (CK, CAS, OIL, and STA). The average lengths of the bacterial and fungal sequences were 426 bp and 177 bp, respectively (Supplementary Tables S1, S2). The coverage rates of both types of sequences were greater than 0.9974 (Supplementary Tables S3, S4), indicating that the sequencing results reflected the true state of the microbial community structure in the sample.

**Figure 3:**
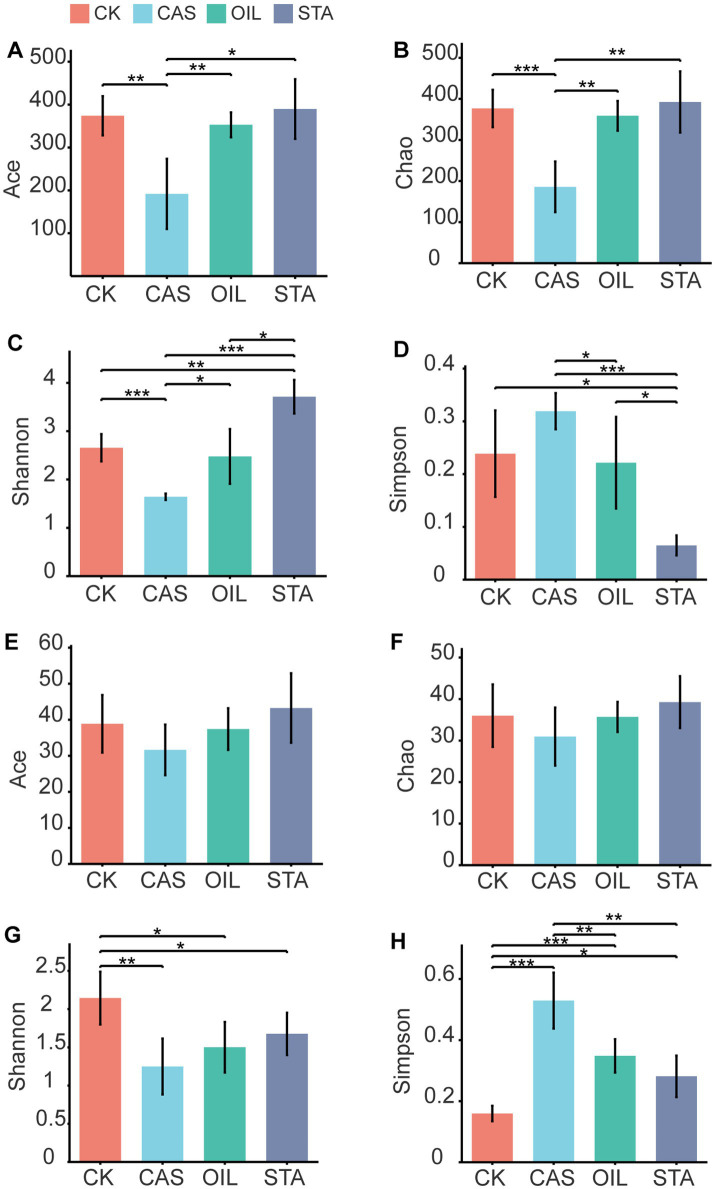
Alpha-diversity of bacterial and fungal communities in the gut of BSFLs fed different diets. **(A–D)** are the Ace, Chao, Shannon and Simpson indexes of the bacterial community, respectively, and **(E–H)** are the diversity indexes of the fungal community.

Feed nutritional composition significantly affected the alpha-diversity of the BSFLs intestinal microbiota (Figure 3). The abundance and diversity of intestinal bacteria in the STA samples were higher than those in other samples, those of the OIL group were equivalent to those of the CK group, and the abundance and diversity of bacteria in the CAS samples were significantly lower than those in the CK and other samples. The diversity of fungi in the STA, OIL and CAS groups was significantly lower than that of the CK group. The richness and diversity of fungi in the CAS group were the lowest.

### Microbial community composition

**Figure 4:**
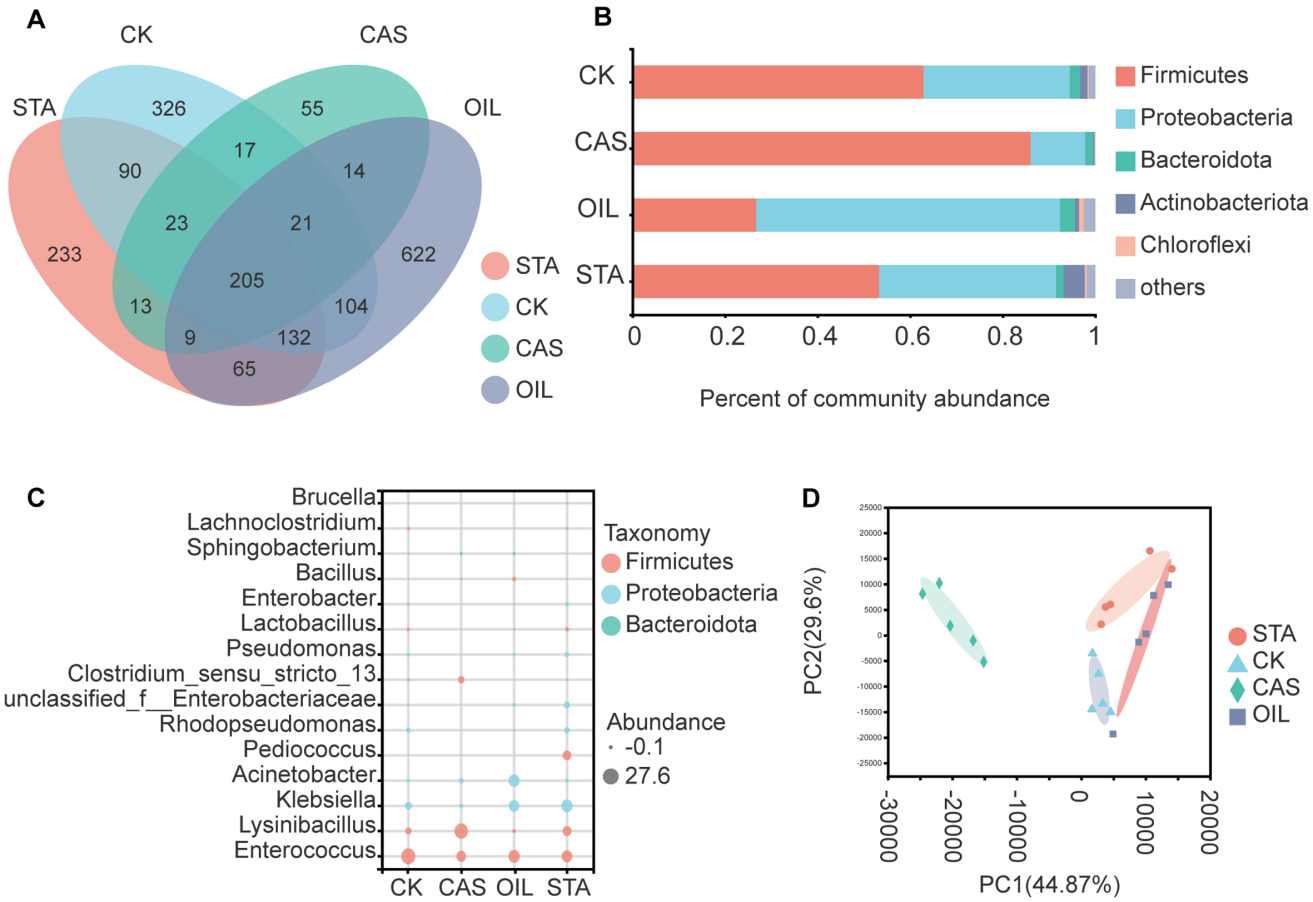
Bacterial community in the gut of BSFLs fed different diets. **(A)** Venn diagram demonstrating the overlap of OTUs. **(B)** Relative abundance of bacterial genera in different samples. **(C)** Bubble plot of bacterial abundance at the genus level. **(D)** Principal component analysis of bacterial communities. The values on axes 1 and 2 are the percentages that can be explained by the corresponding axis. The cluster analysis used the Bray–Curtis distance and complete-linkage method.

Two hundred and five bacterial OTUs were common across all substrate types. The OIL group harbored 622 unique bacterial OTUs, followed by the CK, STA and CAS groups with 326, 233 and 55 unique OTUs, respectively (Figure 4A). The intestinal bacteria of BSFLs were mainly composed of Firmicutes and Proteobacteria. The nutritional composition of the diet significantly affected the bacterial composition at the phylum level. The relative abundance of Firmicutes increased and that of Proteobacteria decreased in the CAS samples, while the OIL group showed the opposite result (Figure 4B). Figure 4C is a bubble diagram of bacterial genus abundance in the BSFLs gut. Compared with the CK group, the abundance of *Enterococcus*, *Klebsiella* and *Rhodopseudomonas* decreased in the CAS group, while that of *Lysinibacillus* and *Clostridium_ sensu_ Stricto_13* increased. The abundance of *Klebsiella*, *Acinetobacter* and *Bacillus* in the OIL group increased, while that of *Enterococcus*, *Lysinibacillus*, *Rhodopseudomonas* and *Clostridium* decreased. The abundance of *Lysinibacillus*, *Klebsiella*, *Pediococcus*, unclassified_f__Enterobacteriaceae, and *Escherichia_Shigella* in the STA group increased and that of *Enterococcus* decreased. In the bacterial principal component analysis results at the OTU level (Figure 4D), the two principal coordinates PC1 and PC2 explain 44.87 and 29.6% of the data changes, respectively. There were obvious differences in the intestinal bacterial composition of the four groups fed different diets, which were clustered separately. PC1 completely separated the CAS group from the other samples, indicating that the bacterial community compositions of the CAS group and the other three groups were quite different. The samples of the OIL group were relatively scattered, indicating the higher diversity of this group. The above results show that the nutritional composition significantly affected the intestinal bacterial community of BSFLs, especially CAS.

**Figure 5:**
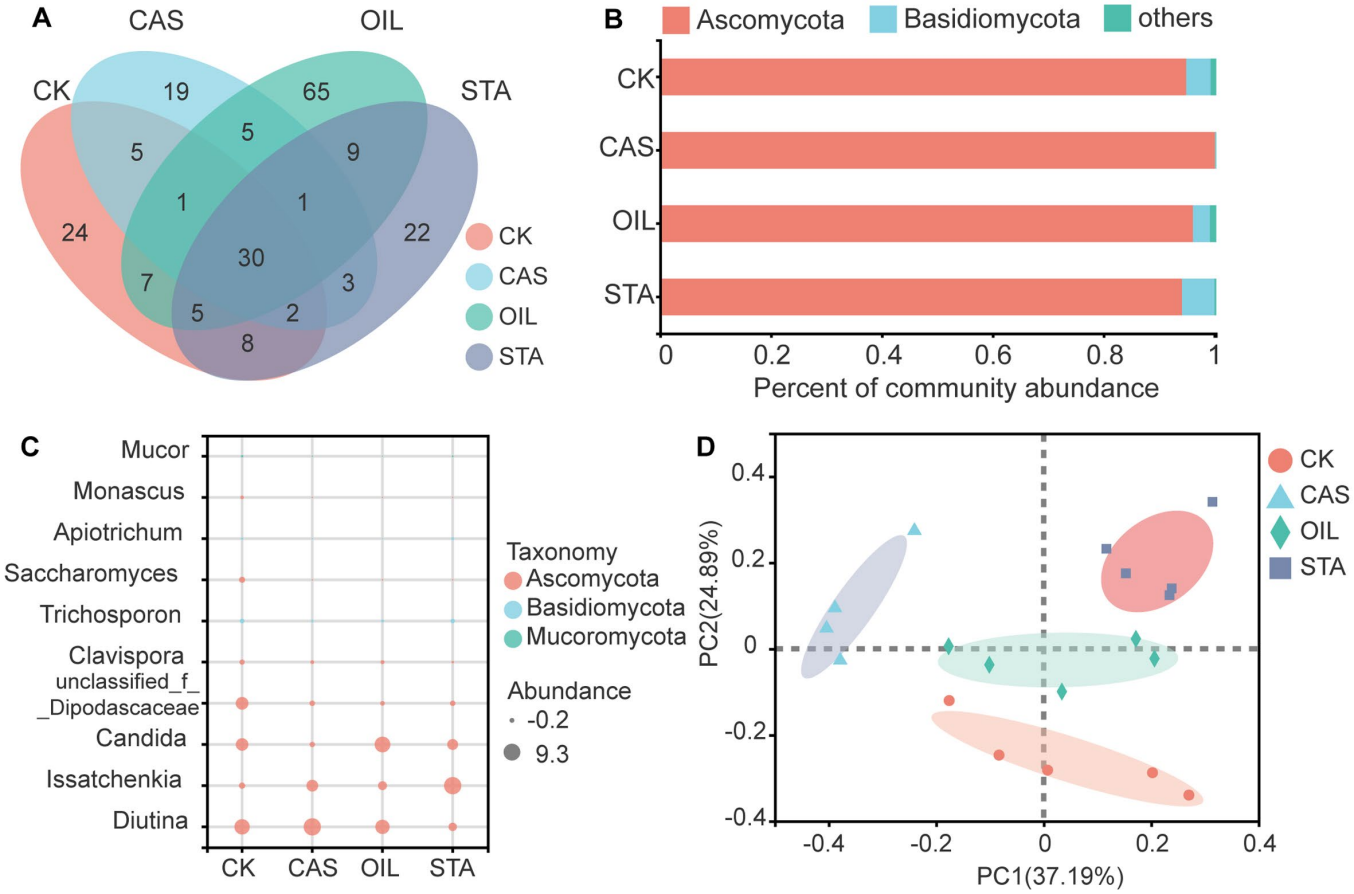
Fungal community in the gut of BSFLs fed different diets. **(A)** Venn diagram demonstrating the overlap of OTUs. **(B)** Relative abundance of fungal genera in different samples. **(C)** Bubble plot of fungal abundance at the genus level. **(D)** Principal component analysis of fungal communities. The values on axes 1 and 2 are the percentages that can be explained by the corresponding axis. The cluster analysis used the Bray–Curtis distance and complete-linkage method.

The composition of the intestinal fungal community of BSFLs was also studied (Figure 5). Thirty fungal OTUs were common across all substrate types. The OIL group harbored 65 unique fungal OTUs, followed by the CK, STA and CAS group, with 24, 22 and 19 unique OTUs, respectively (Figure 5A). The intestinal fungal community of BSFLs was mainly composed of Ascomycota and Basidiomycota. The nutritional composition of the diet affected the fungal composition at the phylum level. The relative abundance of Ascomycota in the CAS samples increased, while that of Basidiomycota decreased. The OIL samples were the most similar to the CK samples (Figure 5B). At the genus level, *Diutina*, *Issatchenkia* and *Candida* were the dominant core fungi in the BSFLs gut. The relative abundance of *Diutina* in the CAS samples was the highest and was much higher than that in the other three samples; the abundance of *Issatchenkia* increased, and that of *Candida* decreased. The fungal composition of the OIL samples at the genus level was most similar to that of the CK samples. In the STA samples, the abundance of *Diutina* and *Candida* decreased, while that of *Issatchenkia* increased. The levels of unclassified-f-Dipodascaceae, *Clavispora*, *Trichosporon*, *Saccharomyces*, and *Monascus* in the CK samples were much higher than those in the other three samples (Figure 5C). The PCoA results for the intestinal fungal community of BSFLs with different nutritional components are shown in Figure 5D. In addition to the scattered OIL samples, the other three samples were clustered separately and dispersed in different quadrants, indicating that the nutritional composition significantly affected the community structure of the intestinal fungi of BSFLs.

### Correlation analysis of environmental factors

**Figure 6:**
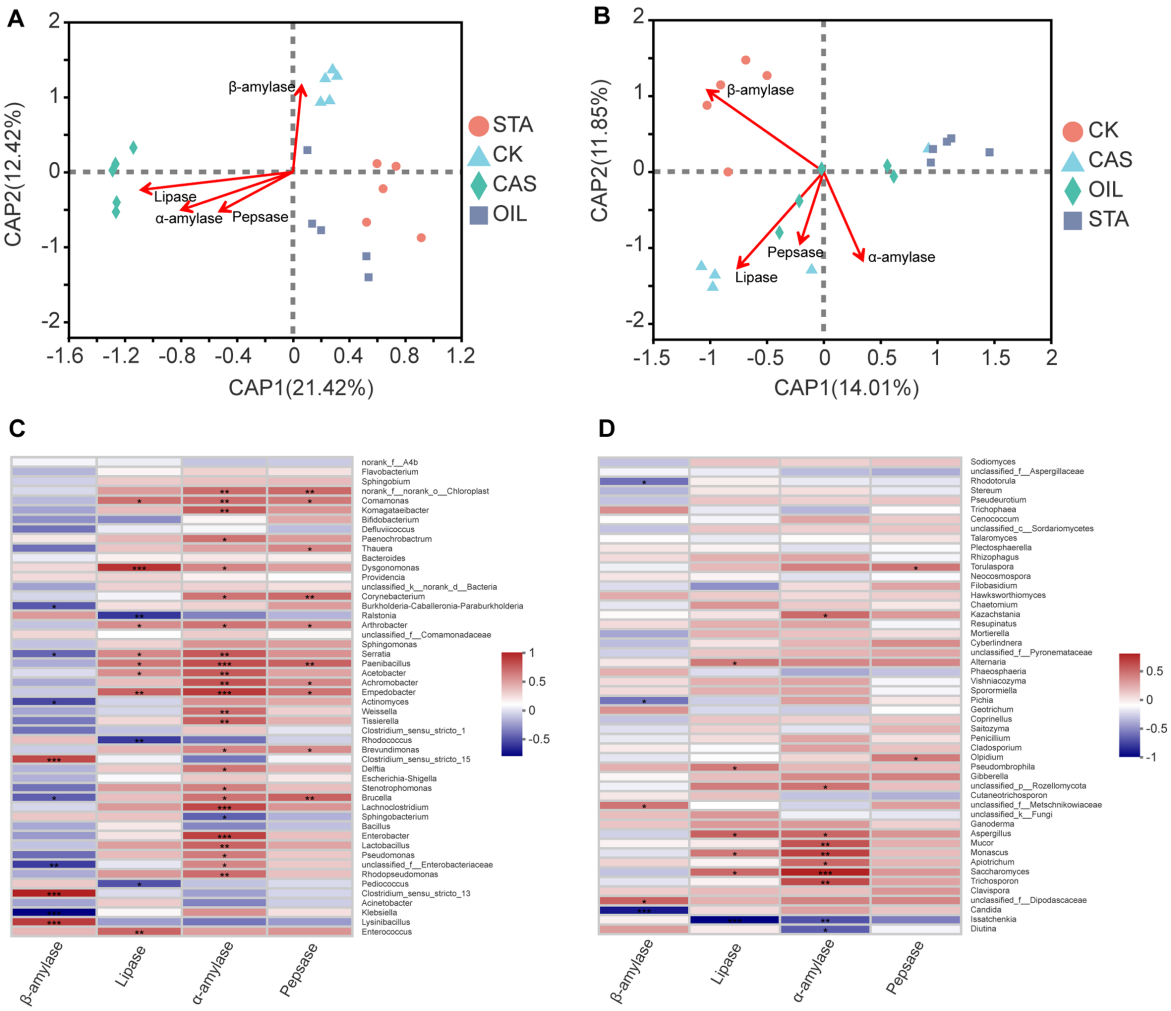
Correlation analysis between the microbial community and digestive enzymes of BSFLs under different nutritional conditions. Distance-based redundancy analysis (db-RDA) biplot of **(A)** bacterial and **(B)** fungal communities with digestive enzymes. Correlation heatmap of the top 50 **(C)** bacterial genera, **(D)** fungal genera and digestive enzymes. *R* values are shown in different colors, and the right side of the legend shows the color ranges of different *R* values. *0.01 < *p* ≤ 0.05, **0.001 < *p* ≤ 0.01, ***p ≤ 0.001.

Environmental factor association analysis (Figure 6) showed that intestinal digestive enzyme activity was closely related to the community of intestinal bacteria and fungi (Supplementary Tables S5 S6). In the core dominant bacterial community, the abundance of *Enterococcus* was strongly positively correlated with lipase activity, the abundance of *Lysinibacillus* was strongly positively correlated with *β*-amylase activity, and the abundance of *Klebsiella* was strongly negatively correlated with *β*-amylase activity. In addition, the abundances of 24 of the top 50 bacterial genera were positively correlated with *α*-amylase activity, and the abundances of 10 genera were positively correlated with pepsase activity. In the dominant fungal communities, there was a negative correlation between the abundances of *Diutina and Issatchenkia* and α-amylase activity, a strong negative correlation between the abundance of *Issatchenkia* and lipase activity, and a strong negative correlation between the abundance of *Candida* and *β*-amylase activity. In addition, among the top 50 fungal genera, the abundances of 8 were positively correlated with *α*-amylase activity, the abundances of 2 were positively correlated with *β*-amylase activity, the abundances of 2 were positively correlated with pepsase activity, and the abundances of 5 were positively correlated with lipase activity.

## Discussion

Previous studies have shown that different food substrates can change the morphology and function of the intestinal tract of BSFLs, including digestive enzyme activity (Bonelli et al., 2020). The researchers compared the effects of a standard diet for dipteran larvae and a vegetable mix diet on the intestinal enzyme activity of BSFLs and found that the chymotrypsin activity of the latter increased significantly, and the α-amylase activity decreased significantly in the posterior part of the intestine, while lipase was not detected (Bonelli et al., 2020). In this study, wheat bran was used as the basic food substrate, and its nutrient components were changed by adding casein, oil and starch. The results showed that the digestive enzyme activities of BSFLs were significantly different under different diets, which was consistent with previous reports. Interestingly, except for β-amylase, the activities of the other digestive enzymes decreased to different degrees under high-protein, high-fat and high-starch diets. These findings indicated that although BSFLs are omnivorous, a balanced diet is more conducive to improving their digestive ability. During the feeding process, the diet should be adjusted reasonably to avoid serious nutritional imbalance. In addition, chymotrypsin and trypsin were not detected, which was inconsistent with the results of other researchers (Bonelli et al., 2020), possibly due to the different genetic backgrounds and diets of the experimental insects.

Although the CK group had higher digestive enzyme activity, its individual insect weight was the lowest. This may be attributed to the low nutrient content of the CK group, as BSFLs need to accumulate large amounts of fat and protein (Rawski et al., 2020). Therefore, the larvae and pupae of the OIL group were the heaviest and were significantly heavier than those of the control group. The STA group and CAS group ranked second, but their weights were not significantly different from those of the CK group. The above results again prove that low-fat feed has a negative impact on the growth of BSF in the whole larval stage and prepupa/pupa stage (Bellezza Oddon et al., 2022). Barragan-Fonseca et al. (2019) found that individual pupal weight showed a strong linear correlation with the carbohydrate content and, to a lesser degree, with the protein content, which is not consistent with the results of this study. Gobbi et al. (2013) found that the mortality rate of black water fly larvae fed a meat meal diet was approximately 60%, which was much higher than that of the larvae fed hen feed and mixed feed. However, Barragan-Fonseca et al. (2019) speculated that the survival rate of BSFLs was not affected by the protein or carbohydrate content. In our experimental results, the high-fat diet group had the highest survival rate, and the high-protein diet group had the lowest survival rate. The inconsistency in the results can be attributed to various factors, including the genetic background, diet, and culture conditions of BSF.

In general, the microbiota in the diet strongly affects the gut microbiota of BSFLs, and many studies have proven this in terms of bacteria (Jeon et al., 2011; Bruno et al., 2019; Ao et al., 2021). In contrast, fungi are rarely mentioned in studies of the intestinal microbiota, but they have received increasing attention in recent years. Varotto Boccazzi et al. (2017) proved that the type of substrate was associated with the difference in the intestinal fungal community of BSFLs. Tanga et al. (2021) observed a significant impact of diet on fungal microbiota richness, diversity, and evenness. Although the diet is considered to be an important source of bacteria (Jiang et al., 2019), the interactions of various abiotic and biological factors can also affect the intestinal microbiota of larvae (Wynants et al., 2019). In this study, all diets were sterilized, and the culture process was also conducted in a sterile environment. The influence of the microbiota in the diet was excluded, and only the levels of protein, oil and starch in the diet were changed. The purpose was to study the effect of dietary nutritional composition on the gut microbiota of BSF. The results showed that the diversities of bacteria and fungi were consistent, showing the trend STA > OIL > CAS. Feeding BSF a diet with a high casein content led to a decrease in the diversity and richness of their intestinal bacterial and fungal communities. High oil content did not significantly change the bacterial diversity but reduced the fungal diversity. High starch content increased the bacterial diversity and reduced the fungal diversity. These results indicated that the composition of dietary nutrition significantly affected the intestinal microbial community structure of BSFLs.

Many researchers believe that the intestinal microbiota of BSFLs is greatly affected by different nutrient sources (Jeon et al., 2011), while others believe that BSFLs have a conserved microbiota (Klammsteiner et al., 2020; Shelomi et al., 2020). Most research reports have indicated that Proteobacteria, Firmicutes and Bacteroidetes are the main bacteria in the intestinal tract of BSF, although their relative abundance varies with food type. Interestingly, Firmicutes and Proteobacteria were the main bacterial groups in the samples in this study, and the relative abundance of Bacteroidetes was low. In previous studies, Bacteroidetes often accounted for a low proportion of the microbiota, and the diet types included rice, chicken manure and fish meat (Jeon et al., 2011; Bruno et al., 2019; Ao et al., 2021), with no regular trend observed. In this study, wheat bran was the main dietary component, and the abundance of Bacteroidetes in the OIL group was the highest, which confirmed the view that Bacteroidetes was related to fat degradation.

Klammsteiner et al. (2020) speculated that *Actinomyces*, *Dysgonomonas* and *Enterococcus* were the core members of the gut community because they were stably present in most samples, regardless of whether the diet was composed of chicken feed, fruit/vegetables or grass cuttings. A stable autochthonous microbiota was conducive to providing tools for degrading a wide range of substrates. In this study, *Enterococcus, Lysinibacillus* and *Klebsiella* were the dominant bacteria and also the core bacteria in the four groups of samples because they were present in all the samples at high abundance. *Enterococcus* is a normal microorganism inhabiting human and animal intestines. However, ectopic parasitism by *Enterococcus* can lead to respiratory tract infection, wound infection and sepsis (Iimura et al., 2020; Maleb et al., 2020; Zsolt and Aqeel, 2021), and *Enterococcus* has developed resistance to a variety of antibiotics (Mayoral-Terán et al., 2020). Therefore, breeders should avoid contact with live larvae of BSF in wounds to prevent infection. *Lysinibacillus* is a genus of environmental gram-positive bacteria that is generally nonpathogenic (Xu et al., 2015; Jin et al., 2017). *Lysinibacillus fusiformis* was previously isolated from the eggs of a BSF colony, and it could dominate the larval microbiota and increase larval weight and survival (Schreven et al., 2021). However, the mortality of CAS in this study was the highest among the four groups (Figure 6), although *Lysinibacillus* was the dominant species in the CAS samples, with much higher abundance in the CAS samples than in the other three samples. The reason for this result is not yet clear. *Klebsiella* strains have become a major clinical and public health threat worldwide (Dong et al., 2022), infecting a variety of animals and causing digestive diseases (Kaur et al., 2018). Therefore, it is necessary to sterilize BSFLs by cooking or drying when using them as feed. In addition, *Acinetobacter* is widely distributed and an important biodegrader of petroleum hydrocarbons. It can secrete lipase to decompose triacylglycerol to fatty acids and glycerol for use by cells (Snellman and Colwell, 2004). This explains why *Acinetobacter* was the dominant bacterial genus (28.8%) in the OIL samples in our experiment and was present at much higher abundance in the OIL samples than in the other three samples.

The fungal communities of BSFLs are highly substrate dependent (Varotto Boccazzi et al., 2017; Tanga et al., 2021; Zhang et al., 2021). Varotto Boccazzi et al. (2017) found that the influence of diet on the composition of the fungal community in the BSFLs gut was so great that no OTU common to all experimental groups was detected. *Pichia* was the most abundant genus associated with larvae fed on vegetable waste, whereas *Trichosporon*, *Rhodotorula* and *Geotrichum* were the most abundant genera in larvae fed on chicken feed only (Varotto Boccazzi et al., 2017). In BSFLs fed chicken manure, *Penicillium* and *Aspergillus* were the main fungi in the gut (Zhang et al., 2021). *Diutina*, *Issatchenkia* and *Candida* were the dominant fungi in the samples of this study. *Diutina* was the dominant fungal genus in all the experimental samples, and its relative abundance in the CAS samples was much higher than that in the other three samples. Further analysis showed that the main species of *Diutina* is *Diutina rugosa*, which is a kind of yeast frequently studied in this genus and is mainly used to produce lipase (de Freitas et al., 2021), being widely used in the chemical, food, energy, and environmental protection industries and other fields (Ali et al., 2017; Sun et al., 2021). *D. rugosa* can also be occasionally found in the environment and the intestines of livestock and poultry. Previous studies confirmed that *D. rugosa* SD-17 improved the growth and regulated the immunity of chickens, so it could be optimized as a feed additive for livestock and poultry to play the role of probiotic (Wang et al., 2019). *Issatchenkia* had the highest abundance in the STA samples. *Issatchenkia* species are unicellular fungi with two metabolic modes: oxidation and fermentation. Some strains have been screened for citric acid degradation and alcohol brewing (Eun et al., 2019; Liu et al., 2021). Candida species are symbiotic and invasive fungi (Lim et al., 2012) with strong pathogenicity and a mortality rate of up to 70%. Their clinical manifestations are fungemia and skin mucosal lesions (Figueira et al., 2020). Candida species can produce hemolysins *in vitro* and exhibit several virulence-related phenotypes, such as adherence, biofilm formation and the secretion of hydrolytic enzymes that cause host cell damage and animal diseases (Silva et al., 2012). The results of this study show that increasing the oil content in the diet will significantly increase the proportion of Candida in the intestinal flora of BSF. Therefore, in the process of BSF breeding, it is necessary to adjust the nutrition ratio in the diet and control the amount of Candida in the gut to reduce the risk of infection to workers and feeding animals. In addition to the above 3 genera, CK also enriched unclassified_f_Dipodascaceae, *Clavispora*, *Trichosporon*, *Saccharomyces*, *Monascus* and *Mucor*. *Trichosporon* species can degrade mycotoxins such as ochratoxin A and zearale-none (Schatzmayr et al., 2006) and inhibit Candida growth. *Monascus* species produce polyketides (Jůzlová et al., 1996). *Saccharomyces* species produce several compounds, mainly including polyketides, terpenoids, and amino acid derivatives (Wang et al., 2021). The possible functions of these secondary metabolites include antibiotic, antifungal, cytostatic or natural insecticide activities, which may help BSFLs resist pathogens and improve survival.

It was reported that feed supplemented with *Enterococcus faecalis* significantly increased the activities of protease and lipase in the fish intestine (Allameh et al., 2017). Interestingly, in this study, the relative abundance of *Enterococcus* in the CK group was higher than that in the treatment groups, and the CK group also showed higher protease and lipase activities (Figures 1C,D, 4C). The correlation analysis of environmental factors showed that the abundance of *Enterococcus* was strongly positively related to lipase activity. Fat is an important means of energy storage in BSFLs, while lipids can be decomposed in the larval gut to free fatty acids or mono- and diglycerides for absorption by gut cells and use in larval metabolism (Carvalho et al., 2012). The abundance of *Enterococcus* was also positively correlated with amylase and pepsin activities, although not significantly. Thus, microbes from the genus *Enterococcus* might be important for lipid and protein conversion in the gut of BSFLs. In addition, *Lysinibacillus* abundance and β-amylase activity also showed a very significant positive correlation. Beta-amylases are mostly produced by plants, as well as some gram-positive spore-forming bacteria such as *Bacillus* spp. (Ajayi and Fagade, 2006). Intestinal bacteria contribute to the nutrition of insects (Engel and Moran, 2013). Fungal sources of α-amylase are confined to terrestrial isolates, mostly to *Aspergillus* species and a few species of *Penicillium* (Sundarram and Murthy, 2014). *Aspergillus* species have strong abilities to synthesize and secrete different enzymes, which could play a critical role in starch saccharification (Oda et al., 2006; Zheng et al., 2011). In this study, the abundance of *Aspergillus* was positively correlated with α-amylase, lipase and pepsase activities. In addition, some species of *Monascus* and *Mucor* can also produce α-amylase (Mohapatra et al., 1998; Yoshizaki et al., 2010; Tallapragada et al., 2017), and the abundances of both of these genera and several other genera showed a significant positive correlation with amylase activity in this study (Figure 6D). However, except for amylase, the abundances of most fungal genera did not show a significant correlation with the activities of the other three enzymes. In contrast, bacteria are more closely related to digestive enzymes than fungi.

Based on the theory of microecology, insect relying on gut microbes provides a variety of digestive enzymes, to complete its food digestion, nutrient absorption and metabolism (Liang et al., 2015). The composition and structure of microbial are dynamic, which can be varied with changing nutrient availability, physiological environments, and the proximity to other organisms (McKillip et al., 1997; Dillon et al., 2005). The production of enzymes by microorganisms as well as the enzyme yield depends on the nutritional factors especially carbon and nitrogen sources (Niyonzima and More, 2014). Therefore, in this study, different diets provided different carbon and nitrogen sources for the intestinal microbes of the BSFL, which affected the composition of the microbial community (Figures 3–6) and its ability to produce enzymes, which was reflected in the difference of digestive enzyme activity in the intestinal tract (Figure 1) and larval performance (Figure 2) of the BSFL.

## Data availability statement

The datasets presented in this study can be found in online repositories. The names of the repository/repositories and accession number(s) can be found in the article/Supplementary material.

## Author contributions

JZ, XZ, WT, and YL designed the study. GC, KZ, JP, and Xin Yuan performed experiments. XS, LJ, and HZ performed bioinformatics and statistical analyses. GC, Xin Yu, and JW wrote the manuscript. All authors contributed to the article and approved the submitted version.

## Funding

This work was supported by the Key Research and Development Plan of Shandong Province (2022CXPT022), Shandong Province Poultry Industry Technology System (SDAIT-11-10), Natural Science Foundation of Shandong Province (ZR2021QC187, ZR2021QC167, ZR2020KC028 and ZR2020QC227), Major Science and Technology Innovation Project of Shandong Province (2017GGH5129), Cooperation Project of University and Local Enterprise in Yantai of Shandong Province (2021XDRHXMXK23), Key Research and Development Plan of Yantai (2021XDHZ076 and 2021YT06000060), and Shandong Agricultural Major Applied Technology Innovation Project (to XZ).

## Conflict of interest

The authors declare that the research was conducted in the absence of any commercial or financial relationships that could be construed as a potential conflict of interest.

## Publisher’s note

All claims expressed in this article are solely those of the authors and do not necessarily represent those of their affiliated organizations, or those of the publisher, the editors and the reviewers. Any product that may be evaluated in this article, or claim that may be made by its manufacturer, is not guaranteed or endorsed by the publisher.

## Footnotes

^1^ www.i-sanger.com

^2^ http://rdp.cme.msu.edu/
