# Supplementary material for: Feed nutritional composition affects the intestinal microbiota and digestive enzyme activity of black soldier fly larvae
Source: Front Microbiol. 2023 May 24;14:1184139. doi: 10.3389/fmicb.2023.1184139 (PMC10244541; doi:10.3389/fmicb.2023.1184139)
Supplement: Supplementary file 1 [file Table_1.DOCX]

Table S1 Statistics of the trimmed sequences of the bacterial communities

| Sample\Info | Seq_num | Base_num | Mean_length | Min_length | Max_length |
| --- | --- | --- | --- | --- | --- |
| CK_1 | 52941 | 22498172 | 425 | 220 | 442 |
| CK_2 | 51826 | 22118287 | 427 | 220 | 441 |
| CK_3 | 51866 | 21958702 | 423 | 202 | 431 |
| CK_4 | 50884 | 21665714 | 426 | 264 | 432 |
| CK_5 | 57110 | 24261733 | 425 | 204 | 441 |
| CAS_1 | 57874 | 24686419 | 427 | 271 | 431 |
| CAS_2 | 60359 | 25735692 | 426 | 219 | 517 |
| CAS_3 | 59306 | 25310757 | 427 | 220 | 512 |
| CAS_4 | 57511 | 24320531 | 423 | 220 | 431 |
| CAS_5 | 59748 | 25446516 | 426 | 220 | 469 |
| OIL_1 | 51654 | 22127057 | 428 | 220 | 432 |
| OIL_2 | 48766 | 20818103 | 427 | 220 | 463 |
| OIL_3 | 55954 | 23911408 | 427 | 220 | 441 |
| OIL_4 | 58847 | 25018801 | 425 | 202 | 432 |
| OIL_5 | 57962 | 24815603 | 428 | 220 | 437 |
| STA_1 | 59091 | 24893354 | 421 | 219 | 489 |
| STA_2 | 55413 | 23539445 | 425 | 219 | 447 |
| STA_3 | 56062 | 23995573 | 428 | 216 | 434 |
| STA_4 | 36131 | 15474236 | 428 | 265 | 431 |
| STA_5 | 53801 | 22723554 | 422 | 220 | 431 |

Table S2 Statistics of the trimmed sequences of the fungal communities

| Sample\Info | Seq_num | Base_num | Mean_length | Min_length | Max_length |
| --- | --- | --- | --- | --- | --- |
| CK_1 | 99844 | 16187593 | 162 | 140 | 446 |
| CK_2 | 68398 | 13711771 | 200 | 140 | 440 |
| CK_3 | 54452 | 13455394 | 247 | 140 | 512 |
| CK_4 | 111253 | 18075205 | 162 | 140 | 484 |
| CK_5 | 75865 | 13649845 | 180 | 140 | 443 |
| CAS_1 | 115915 | 20420269 | 176 | 140 | 284 |
| CAS_2 | 120147 | 18951757 | 158 | 140 | 533 |
| CAS_3 | 100040 | 14868818 | 149 | 140 | 441 |
| CAS_4 | 147940 | 21801994 | 147 | 140 | 284 |
| CAS_5 | 93188 | 14004232 | 150 | 140 | 439 |
| OIL_1 | 80593 | 15772678 | 196 | 140 | 442 |
| OIL_2 | 91924 | 15594646 | 170 | 140 | 439 |
| OIL_3 | 104714 | 17544720 | 168 | 140 | 437 |
| OIL_4 | 91344 | 17570978 | 192 | 140 | 481 |
| OIL_5 | 89948 | 16224319 | 180 | 140 | 438 |
| STA_1 | 106853 | 18471459 | 173 | 140 | 438 |
| STA_2 | 91013 | 16638200 | 183 | 140 | 485 |
| STA_3 | 106997 | 19755571 | 185 | 140 | 439 |
| STA_4 | 141157 | 26267154 | 186 | 140 | 437 |
| STA_5 | 98488 | 17195215 | 175 | 140 | 444 |

Table S3 Bacterial diversity indexes

| Sample | Shannon | Simpson | Ace | Chao | Coverage |
| --- | --- | --- | --- | --- | --- |
| CK_1 | 2.7432 | 0.2542 | 446 | 449 | 0.9984 |
| CK_2 | 2.2480 | 0.3171 | 365 | 373 | 0.9983 |
| CK_3 | 3.0053 | 0.1303 | 353 | 353 | 0.9987 |
| CK_4 | 2.5221 | 0.3104 | 382 | 379 | 0.9987 |
| CK_5 | 2.7365 | 0.1790 | 322 | 327 | 0.9987 |
| CAS_1 | 1.6830 | 0.2900 | 136 | 166 | 0.9991 |
| CAS_2 | 1.6219 | 0.3694 | 241 | 244 | 0.9984 |
| CAS_3 | 1.7070 | 0.2871 | 142 | 138 | 0.9992 |
| CAS_4 | 1.5357 | 0.3354 | 126 | 122 | 0.9992 |
| CAS_5 | 1.6380 | 0.3120 | 312 | 256 | 0.9979 |
| OIL_1 | 2.1043 | 0.2202 | 330 | 327 | 0.9979 |
| OIL_2 | 2.6364 | 0.1731 | 330 | 340 | 0.9986 |
| OIL_3 | 2.5191 | 0.2055 | 357 | 357 | 0.9984 |
| OIL_4 | 3.2974 | 0.1401 | 676 | 673 | 0.9976 |
| OIL_5 | 1.8056 | 0.3665 | 392 | 409 | 0.9974 |
| STA_1 | 4.0232 | 0.0495 | 410 | 411 | 0.9990 |
| STA_2 | 3.3349 | 0.0858 | 311 | 310 | 0.9994 |
| STA_3 | 1.5891 | 0.3191 | 183 | 163 | 0.9990 |
| STA_4 | 1.3562 | 0.4543 | 105 | 122 | 0.9993 |
| STA_5 | 3.7669 | 0.0578 | 446 | 455 | 0.9988 |

Table S4 Fungal diversity indexes

| Sample | Shannon | Simpson | Ace | Chao | Coverage |
| --- | --- | --- | --- | --- | --- |
| CK_1 | 2.1265 | 0.1785 | 49 | 43 | 1.0000 |
| CK_2 | 2.4453 | 0.1361 | 29 | 27 | 1.0000 |
| CK_3 | 2.4255 | 0.1378 | 35 | 34 | 1.0000 |
| CK_4 | 1.5896 | 0.3122 | 45 | 44 | 1.0000 |
| CK_5 | 2.1184 | 0.1825 | 37 | 31 | 1.0000 |
| CAS_1 | 1.8689 | 0.2442 | 41 | 39 | 1.0000 |
| CAS_2 | 1.2237 | 0.4028 | 32 | 32 | 1.0000 |
| CAS_3 | 0.9165 | 0.6223 | 34 | 34 | 1.0000 |
| CAS_4 | 1.075 | 0.5511 | 31 | 30 | 1.0000 |
| CAS_5 | 1.1515 | 0.5371 | 21 | 20 | 1.0000 |
| OIL_1 | 2.0378 | 0.2709 | 77 | 77 | 1.0000 |
| OIL_2 | 1.3905 | 0.3472 | 32 | 32 | 1.0000 |
| OIL_3 | 1.136 | 0.4169 | 36 | 36 | 0.9999 |
| OIL_4 | 1.4342 | 0.3768 | 44 | 41 | 1.0000 |
| OIL_5 | 1.4918 | 0.3262 | 0 | 34 | 1.0000 |
| STA_1 | 1.5443 | 0.3132 | 42 | 42 | 1.0000 |
| STA_2 | 1.8498 | 0.2336 | 31 | 30 | 1.0000 |
| STA_3 | 1.9877 | 0.2035 | 38 | 38 | 1.0000 |
| STA_4 | 1.2736 | 0.3772 | 55 | 39 | 0.9999 |
| STA_5 | 1.7118 | 0.2744 | 49 | 47 | 1.0000 |

Table S5 db-RDA at the OTU level for the bacterial community

|  | CAP1 | CAP2 | r2 | p_values |
| --- | --- | --- | --- | --- |
| α-Amylase | -0.8689 | -0.495 | 0.5997 | 0.001 |
| β-Amylase | 0.1145 | 0.9934 | 0.7568 | 0.001 |
| Pepsase | -0.7576 | -0.6527 | 0.3519 | 0.03 |
| Lipase | -0.9745 | -0.2244 | 0.8551 | 0.001 |

Table S6 db-RDA at the OTU level for the fungal community

|  | CAP1 | CAP2 | r2 | p_values |
| --- | --- | --- | --- | --- |
| α-Amylase | 0.4337 | -0.9011 | 0.4697 | 0.007 |
| β-Amylase | -0.6997 | 0.7145 | 0.7787 | 0.001 |
| Pepsase | -0.0082 | -1 | 0.238 | 0.099 |
| Lipase | -0.4092 | -0.9124 | 0.5141 | 0.002 |
